# The Complementary Role of Morphology in Understanding Microglial Functional Heterogeneity

**DOI:** 10.3390/ijms26083811

**Published:** 2025-04-17

**Authors:** Sânziana Godeanu, Bogdan Cătălin

**Affiliations:** 1 Experimental Research Centre for Normal and Pathological Aging, University of Medicine and Pharmacy of Craiova, 200349 Craiova, Romania; sanzianagodeanu@yahoo.com; 2 Department of Molecular Physiology, Center for Integrative Physiology and Molecular Medicine (CIPMM), Building 48, University of Saarland, 66421 Homburg, Germany

**Keywords:** microglia, morphology, transcriptomics, heterogeneity

## Abstract

A search of the PubMed database for publications on microglia reveals an intriguing shift in scientific interest over time. Dividing microglia into categories such as “resting” and “activated” or M1 versus M2 is nowadays obsolete, with the current research focusing on unraveling microglial heterogeneity. The onset of transcriptomics, especially single-cell RNA sequencing (scRNA-seq), has profoundly reshaped our understanding of microglia heterogeneity. Conversely, microglia morphology analysis can offer important insights regarding their activation state or involvement in tissue responses. This review explores microglial heterogeneity under homeostatic conditions, developmental stages, and disease states, with a focus on integrating transcriptomic data with morphological analysis. Beyond the core gene expression profile, regional differences are observed with cerebellar microglia exhibiting a uniquely immune-vigilant profile. During development, microglia express homeostatic genes before birth, yet the bushy appearance is a characteristic that appears later on. In neurodegeneration, microglia alternate between proinflammatory and neuroprotective roles, influenced by regional factors and disease onset. Understanding these structural adaptations may help identify specific microglial subpopulations for targeted therapeutic strategies.

## 1. Introduction

**Figure 1:**
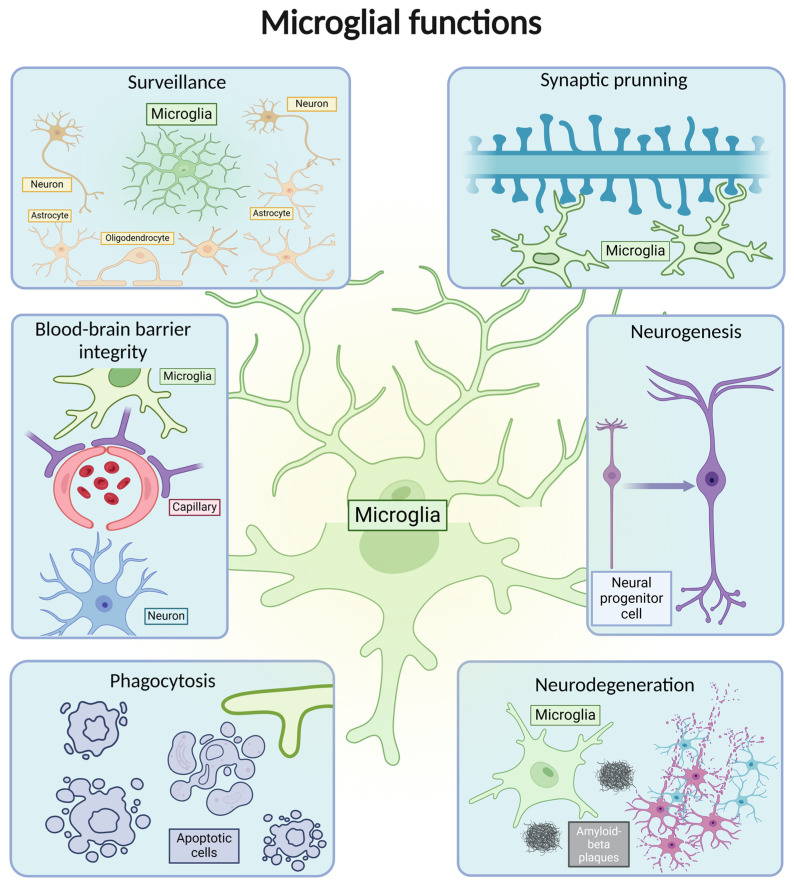
Microglial functions in homeostasis and disease. The figure illustrates a central microglial cell with the surrounding labeled sections representing various functions, such as surveillance, phagocytosis, synaptic pruning, roles in neurodegeneration and neurogenesis, along with maintaining blood–brain barrier integrity. The design depicts representations of both resting and activated states.

Microglia are constantly extending and retracting their processes to monitor the brain’s microenvironment [1,2]. Alongside neurogenesis [3], synaptic pruning [4], and the phagocytosis of developing neurons [5], this continuous surveillance function is considered essential for brain development (Figure 1). In adult life, microglia continue to regulate neurogenesis, particularly in the hippocampus, by pruning newborn cells during critical survival periods [3,6], underlining the physiological importance of these cells across their lifespan. Additionally, upon detecting damage-associated molecular patterns from endogenous sources such as injured neurons or exogenous molecules grouped under the name of pathogen-associated molecular patterns, microglia shift from surveillance to an immune role, performing phagocytosis to clear debris, dead cells, and pathogens [7,8].

Microglia research has exponentially increased over time, with less than 7% of all indexed research published before the year 2000. This increase seems to follow the paradigm changes regarding microglia: from their dynamism, function, and later realization that microglia are a heterogeneous population [9,10]. Factors such as brain region [9,11], age [12,13,14], and disease state [13,15] can all trigger variations in microglia morphology, gene expression, and function (Table 1). Although studying microglia heterogeneity using transcriptomics can provide an in-depth molecular view of gene expression changes, what it does not provide are insights regarding rapidly occurring processes such as the phagocytosis of a dead neuron or movement toward a lesion site [16,17,18]. Likewise, a morphological analysis of microglia generates an incomplete overview. This type of study can describe in detail changes in dynamics [1,19,20], phagocytic capacity [21], branch transformations [12,22,23], and more, but they lack the depth of transcriptional studies.

In the present review, we will focus on the potential of combining these two types of analysis as complementary ways of studying microglia, namely, the integration of morphological and transcriptomic data. Furthermore, we will highlight the possible impediments in interpreting these results, especially due to the heterogeneity of currently existing microglial investigation methods and the lack of standardization.

## 2. Microglia Origin and Population Maintenance

Similar to Kupffer cells (the macrophage equivalents in the liver), Langerhans cells (the skin), osteoclasts (bone), and microglia are regarded as the macrophages of the central nervous system [35]. Due to this fact, for a long time, the idea of a common mesodermal origin was generally accepted [36]. However, it is now established that microglia arise from unique embryonic progenitors that originate from the yolk sac, at least in animal models [37,38]. They colonize the neuroepithelium beginning around embryonic day 9.0 [37,39]. In the human cortex, microglial cells first emerge close to the meninges and choroid plexus near the di-telencephalic fissure as early as 4.5–5 gestational weeks [40,41]. Moreover, a second wave, possibly originating from the vasculature, was observed in periventricular microglia at gestational week 12 [41].

Following the initial colonization, the microglia population remains relatively stable and might be completely renewed every 96 days [42]. The adult microglia numbers are maintained by the proliferation of the progenitors, with a proliferation rate of around 0.5–2.5% [42]. This balance can vary over time, with an increase in apoptosis and a decrease in proliferation being noted immediately postnatally [43]. The microglial reactivity seems to be higher during early development, with the largest numbers of microglial cells being reported around two weeks postnatally. This peak is then followed by a rapid decline to nearly half the maximum level, which then stabilizes, with slight fluctuations into adulthood and aging [12,43].

However, this relatively stable proliferation is overridden in injury and/or disease, where microglia undergo a higher proliferation rate. This phenomenon has been described in all central nervous system lesions from spinal cord injury, where the “microglial scar” formation is considered to facilitate functional recovery [44], to Alzheimer’s disease (AD), where studies on tau protein have shown that by depleting microglia, the propagation of tau is suppressed [45], once again reflecting the complexity of microglia function.

Interestingly enough, in order to maintain cell density, bone marrow-derived microglia can migrate into the brain. This process seems to be mediated by myeloattractant chemokines (CXCL12, CCL2) and is facilitated by an impaired integrity of the blood–brain barrier, phenomena described in patients that underwent irradiation or chemotherapy [46].

## 3. Methods for Examining Microglial Morphology and Associated Challenges

Microglial morphology was first studied using silver staining [47], followed by the Nissl technique [48] and lectin stains (GS-1, RCA, WGA, and ConA) [49]. Progress was subsequently made in the field of immunohistochemistry with the F4/80 marker being one of the first used [9]. This was followed by the use of additional proteins, including CD40, CD11b, CD80, TREM2, and Iba1, a widely used calcium-binding protein involved in microglial activation and motility [50,51]. The microglial markers, along with their main characteristics, advantages, and limitations, are summarized in Table 2. Double-staining protocols, such as Iba1/CD68 [52] or combinations like Iba1/CD11b/ICAM-1 [53], have also been used to study microglial activation in greater detail. The analysis of microglial–neuron interactions has benefited from using double-staining protocols as well [54,55]. Because the expression of microglial markers changes with activation, one must be careful in choosing them. Markers characteristic of an activated cell can be found, but in very small percentages, at the level of branched microglia, and this phenomenon can induce false results in the study [56]. Furthermore, fixation procedures can have a great impact on microglia morphology, with the direct immersion of the fresh brain in paraformaldehyde fixation solution overnight causing the utmost differences when compared to in-vivo analysis [23].

Research in the field has progressed with the development of transgenic mouse models and the use of either a constitutive or an inducible Cre system for microglia-specific studies [57]. While the constitutive system is extensively used, the inducible CreER system offers the advantage of temporal control over gene manipulation [58]. CX3CR1, Tmem119, and Hexb are just a few examples of Inducible Cre systems that are characterized by different efficiency and specificity rates; the researcher merely has to select the model that best suits the objectives of their experiment [59,60,61,62].

Although there are clear limitations of microglia morphology studies, the intricate relation between microglia morphology and function has generated a need for detailed microglia analysis. As such, microglia in specific brain areas, species, and age groups have been analyzed, and an impressive amount of data has been generated. Initially, manual analysis was considered the gold standard for assessing microglial density and distribution. However, manual/semi-manual analysis has the disadvantages of being time-consuming and does not allow the analysis of large amounts of data, introducing additional subjective variations to the results. To address these challenges, several semi-automatic computational algorithms and programming platforms have been developed. A long-standing example is ImageJ, version 1.54p and its plugins [63]; this was followed in tumor identification by the QuPath software, version 0.5.1 [64,65]. The Imaris software, version 9.31 allows for the reconstruction of microglial surfaces [66], and the 3Dmorph MATLAB-based tool, https://github.com/ElisaYork/3DMorph, accessed on 14 April 2025, was designed specifically for 3D cellular morphology analysis [67].

While increasing detailed arborization has allowed researchers to better classify microglia as being in surveillant or activated states, these methods have also introduced a large number of additional parameters that can be used to characterize microglia morphology. While on the surface, this seems like a better system, by allowing the investigative team to select which parameters to include in the final analysis, the overall process remains largely subjective. This can be, at least partially, one of the reasons why considerable variability is reported across studies, making it difficult to achieve consistent results. Table 3 highlights the most commonly described variables for studying microglial morphology and also their interpretations in the context of health and disease. One might think that by increasing the number of parameters analyzed, they are reducing the risk of error, but this can subsequently lead to a large number of false-positive correlations/differences being reported. To address this issue, some researchers have integrated multiple parameters into composite indices. For example, the Inflammation Index, which incorporates 62 distinct morphological features, has been successfully applied to assess the impact of high-fat diet (HFD) feeding on microglial morphology in CX3CR1-GFP ± mice [68].

**Table 2:** Key microglial markers from early to modern techniques: characteristics, advantages, and limitations. This table illustrates the main microglial markers discussed in the text along with their advantages and limitations, starting from the first methods of microglial identification, such as the ammoniacal silver carbonate staining of Hortega, to those most commonly used today.

Microglia Identification Markers	Characteristics	Merits	Limitations
Ammoniacal silver carbonate staining [69,70]	Developed by Pío del Río-Hortega. It was the first staining protocol to allow visualization of microglia and their differentiation from oligodendrocytes.	Adaptations of this technique have also been used for investigating ciliate protozoan systematics and/or ciliate cortical structure and morphogenesis.	It is limited to early stages of research; lacks detailed morphological analysis.
Nissl technique [48]	It was developed by Franz Nissl. Using this staining protocol, he was the first to identify the rod microglia.	The staining was not only used for microglial identification but also for the visualization of neurons.	The staining does not show cellular morphological details.
Lectin stains [49]	Lectins are carbohydrate-binding proteins that present specificity for a particular carbohydrate.	For microglia, the most intense staining was observed with GS-1, RCA, WGA, and ConA. It is a technically easy and reliable staining method.	The degree of lectin binding depends on the stage of microglial activation, with resting microglia reacting less to lectins.
F4/80 [56]	A cell surface glycoprotein specific to macrophages.	It is expressed in mice (without evidence in humans) and is one of the most specific markers for macrophages and microglia.	Being a macrophage marker, it does not have microglial specificity.
CD40 [71,72]	Represents a member of the tumor necrosis factor receptor family and is involved in immune response.	Useful for studying the activation of microglia in various conditions, including Alzheimer’s disease.	Can also be expressed in other immune cells.
CD11b [50]	Forms a part of complement receptor 3 and is involved in adhesion processes and uptake of complement-coated molecules.	It is expressed both in the activation and the resting state of microglia.	It is not a specific marker for microglia. It is also present on the membranes of leukocytes.
CD80 [73,74]	T-lymphocyte activation antigen CD80; a co-stimulatory molecule for CD28 that is expressed on T cells.	The role of CD80 has mainly been studied in microglia–T cell interactions.	Can also be expressed in other immune cells (antigen-presenting cells, regulatory T cells).
TREM2 [50]	Triggering receptor expressed on myeloid cells 2. Controls toll-like receptor 4 signaling.	It has been studied particularly in Alzheimer’s pathology; its alleles represent a genetic risk factor for the development of AD.	Conflicting data in the literature, with studies showing increases in TREM2 in AD and others showing non-significant changes compared to controls.
Iba1 [75,76]	A calcium-binding protein involved in microglial activation and motility.	It is one of the most commonly used markers for microglia identification. Iba1 stains more microglia phenotypes such as ramified, activated, amoeboid, or dystrophic microglia.	Plays an important role, especially in activated microglia; thus, it does not distinguish between different stages of microglial activation.
CD68 [50]	Cluster of differentiation 68 or macrosialin. It is strongly upregulated during inflammation.	Although it is also expressed to some extent in resting microglia, it is considered a marker of activated phagocytic microglia. Increased expression of this marker has been found in the brains of AD patients.	It can also be identified in infiltrating macrophages.
ICAM-1 [77,78,79]	Intercellular Adhesion Molecule 1 is a cell surface glycoprotein involved in leukocyte extravasation and in the interaction of lymphocytes with antigen-presenting cells.	It is useful in the study of microglial cells in various pathological conditions such as axonal injury, the biology of psychiatric disorders, or in models of Alzheimer’s disease.	It is also expressed in the astrocytes and in the endothelial cells of the human brain.

**Table 3:** Key parameters of microglial morphology and motility. This table summarizes the most commonly used parameters related to microglial morphology and motility, with a focus on how these parameters are calculated and the changes across different age groups and in response to specific experimental conditions. The parameters identified are area surveilled/cell environment area, cell area, soma area/cell body area/cell volume, cytoplasm area, total length of branch tree, total number of processes, mean branch length, total number of primary filaments, total number of secondary filaments, total number of endpoints, and process or soma motility.

Parameter	Exemplification	Research Context	Observations
Area surveilled/cell environment area [9,12,24,27,80,81,82,83]	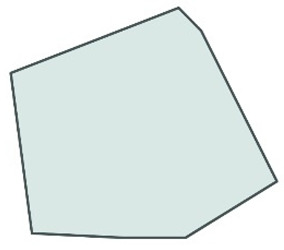	Used to assess microglia morphology across brain regions, upon activation or in association with neuronal activity.	Cerebellar microglia have a smaller surveilled area. During the dark cycle, microglia are more ramified. An increase in microglial domain volume was observed one day following subarachnoid hemorrhage (SAH).
Cell area [12,25]	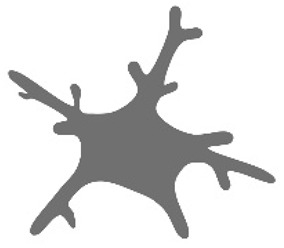	Analyzed in models of brain injury (experimental cerebral ischemia) but also in the normal aging process.	Increases with age. Activation itself increases cell area through soma enlargement. Activated microglia in the cortex have a greater cell area compared to those in the hippocampus.
Soma area/cell body area/cell volume [24,25,27,81,84]	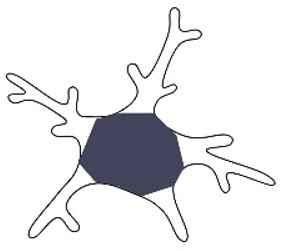	This parameter is used to morphologically determine microglial activation (LPS-induced inflammation, response to laser lesion), as well as to study aging or inter-regional heterogeneity.	Hippocampal microglia have a smaller soma area compared to cortical microglia. Upon treatment with LPS, microglia become more homogeneous than in the control groups. Soma enlargement occurs with age and injury. Rod-like cells have a greater soma area.
Cytoplasm area [81]	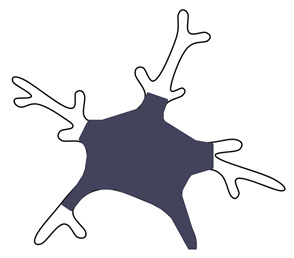	Used to exemplify activation in the context of LPS-induced inflammation but also inter-regional differences, both in pathology and in physiological conditions.	This is the cell body area associated with the cytoplasmic area of the primary ramifications. Cerebellar microglia have a greater cytoplasm area compared to frontal cortex or striatum.
Total length of branch tree [11,12,23,25,54,63,80,85]	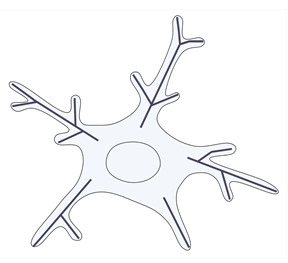	To quantify the arborization in mouse models of Alzheimer’s disease (APP/PS1 Tg mice), after ischemic stroke and reperfusion, after microglia ablation or depletion, or to exemplify the normal aging process or the changes induced by a certain fixation method.	Decreased skeleton length is observed in activated microglia upon injury. Age differences show a sharp postnatal increase, and then a slight decline in older mice. A greater number upon plunge fixation. Rod-like cells have a similar skeleton length to ramified cells. Microglia from the Nac (nucleus accumbens) and SNr (substantia nigra pars reticulata) exhibit greater process lengths.
Total number of processes [12,23,80,86,87]	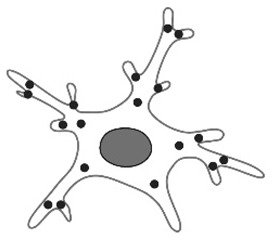	Used to quantify microglial changes due to fixation or aging, as well as in studies involving microglial depletion or mouse models of Alzheimer’s disease (APP/PS1 Tg mice).	Microglia possess fewer branches after PFA perfusion. A drastic increase in the number of branches occurs immediately postnatally. Fewer branches in circumventricular organs (CVOs).
Mean branch length [12,23,80]	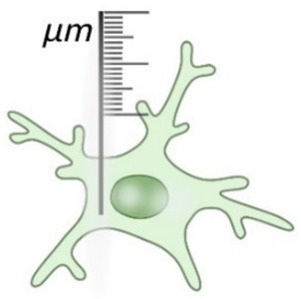	This parameter is used to quantify microglial changes due to fixation or aging or in mouse models of Alzheimer’s disease (APP/PS1 Tg mice).	Old mice possess fewer short branches. Mean branch length is greatest upon PFA perfusion.
Total number of primary filaments [12,23,88,89]	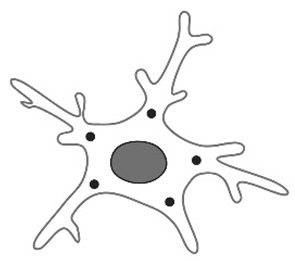	Primary processes are the initial extensions that arise directly from the cell body (soma) of the microglia. Used to study the phenomenon of aging but also in studies that quantify immediately postnatal morphological changes. This parameter also changes according to the fixation method.	Changes are age-related, starting from immediately postnatal, with an increase in the number of primary processes from P5 to P7. Their numbers differ even within the same brain region (e.g., cerebellum).
Total number of secondary filaments [12,23]	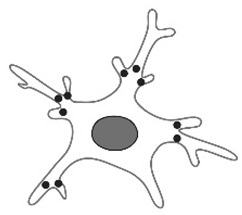	Secondary processes are branches that emerge from the primary processes. Used to quantify microglial changes due to fixation or aging, being part of the parameters that quantify the complexity of the branching pattern.	There is a steep increase immediately postnatally. A change is also observed with the fixation methods.
Total number of end points [12,23,25,54,63,85]	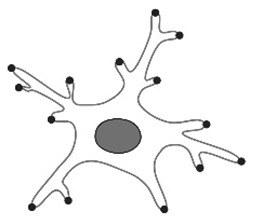	The parameter is more commonly used as the total number of secondary filaments. Used to quantify microglial changes due to fixation or aging, being part of the parameters that quantify the complexity of the branching pattern.	There is a decrease upon injury (in activated microglia). Drastic increase immediately postnatally, followed by a decrease in aged mice. Amoeboid microglia possess the lowest number of endpoints.
Process or soma motility [24,90]	Direction of movement 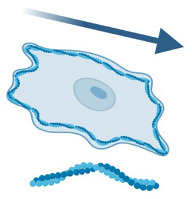	It serves to quantify the microglial response to a pathological stimulus with age, laser injury, or in the context of neurodegenerative diseases (such as in an APPPS1 mouse model of AD).	Process motility significantly decreases with age (possibly affecting surveillance). However, soma movement increases with age, indicating a change in microglia phenotype. A delayed response to injury is observed in aged mice. Microglial motility is impaired in the presence of Aβ plaques when a focal laser lesion is induced.

## 4. Molecular and Cellular Regional Differences in Microglia

As a result of their function in the central nervous system, namely the ability to respond to any stimulus in the microenvironment, microglia are extremely versatile, changing their appearance within minutes after they detect an injury [19]. This heterogeneity has been expected to extend at the spatial level, a phenomenon called spatial heterogeneity [91]. For exemplification and a comprehensive analysis of heterogeneity, the studies presented in the following paragraphs have used rodent models. Although studies dealing with human microglial cells can be found in the literature, they do not reach the complexity of animal models. Moreover, a substantial amount used the TgH (CX3CR1-EGFP) transgenic mouse model [57].

### 4.1. Inter-Regional Morphological Changes

The first indication of microglia heterogeneity was highlighted by quantifying their density (Table 4). Cortical microglia, one of the most studied types, is presented in the form of a cell with a small cell body and a few primary branches, which will dichotomize up to the level of the terminal branches, reaching a complex arborization [12,24]. Variations of this phenotype occur especially in areas where microglia can directly respond to systemic metabolic or inflammatory changes, i.e., those regions of the brain that lack the blood–brain barrier, the so-called “windows of the brain” [92]. Microglia in the pituitary stalk, neurohypophysises are compactly shaped cells with irregular cell bodies and shorter processes [93]. This aspect also characterizes microglia located around the third and fourth ventricles but also in the subventricular zone, where they adopt an amoeboid shape reminiscent of activated microglia [94,95].

At the cellular level, cerebellar microglia also exhibit disparities when compared to cortical microglia [81,96]. They are characterized by lower densities, a larger cytoplasmic area, a smaller surveillance area, and overall reduced ramification [81,96]. The hippocampus has higher microglial densities, exceeding those of the cortex [9]. The degree of ramification and also the soma area are both reduced when comparing hippocampal to cortical microglia, an aspect that is also preserved in case of activation [25]. The basal ganglia areas responsible for motor control have the highest microglial densities [9]. Microglia in the NAc (nucleus accumbens) and SNr (substantia nigra pars reticulata) have a large total process length and a high number of branches, resulting in a high degree of complexity [11].

### 4.2. Intraregional Morphological Changes

The term heterogeneity can be applied when comparing microglia from the same region of the brain. Based on density, the basal ganglia are heterogeneous areas [9]. While the ventral pallidum contains the highest number of microglia, the apical portion of the neostriatum has the lowest number of microglia [9]. The only area with the highest densities close to the cortex is the NAc [11]. Moreover, if the most ramified microglia are found in the NAc and SNr, the microglia in the VTA (ventral tegmental area) and SNc (substantia nigra pars compacta) are sparsely branched and have a reduced surveillance area [11]. Intraregional changes also extend to the cerebellar microglia level, with higher concentrations in the deep cerebellar nuclei and lower concentrations in the molecular layer [9]. The molecular layer is also the one with the fewest microglia [9]. In the hippocampus, microglial density is lower in the CA3 region compared to the CA1 and dentate gyrus, which show the highest density [9].

**Table 4:** Microglial cell density across brain regions in young to adult mice. The table presents the density of microglial cells (cells/mm^3^) in various brain regions compiled from studies focusing on young to adult mice, with values reported alongside the standard error of the mean where available. Only values from studies where the results fell within a reasonable range were included. Studies with mean values deviating by more than a factor of two from others were excluded. In instances where cell density was reported in two-dimensional (2D) formats, values were adjusted to volumetric density by dividing by the slice thickness. Additionally, data extracted from bar plots using Graph Digitizer, version 2.24 were included when necessary to ensure consistency across studies.

Region of the Brain		Density (Cells/mm^3^) ± SEM
Cortical areas	Neocortex general	6500 ± 600 [1]
	Somatosensory cortex	8892 ± 535 [54], 7400 ± 900 [97]
	Frontal cortex	6200 ± 500 [9], 5823 ± 297 [98], 123,60 [81]
	Motor cortex	8000 ± 800 [97]
	Occipital cortex	6200 ± 300 [9], 5589 ± 223 [98]
	Parietal cortex	6124 ± 319 [98], 6900 ± 500 [9]
	Cingulate cortex	5600 ± 500 [9]
	Sensoriomotor cortex	7900 ± 300 [9]
	Visual cortex	7250 [27]
	Auditory cortex	7500 [27]
Basal ganglia	Neostriatum apical	9100 ± 1000 [9]
	Ventral pallidum	16,500 ± 900 [9]
Hippocampus	Dentate gyrus	12,000 ± 700 [9]
	Dorsal hippocampus	5890 ± 280 [99]
	Ventral hippocampus	5460 ± 300 [99]
Cerrebelum	Cerebellar nuclei	7300 ± 400 [9], 5579 ± 793 [89]
	Molecular layer	2200 ± 300 [9], 1387 ± 108 [89]
	Granular layer	3312 ± 343 [89]
	White matter	3981 ± 780 [89]

### 4.3. Transcriptomic Profile Changes

Transcriptomic studies have further highlighted the idea of inter- and intraregional heterogeneity while maintaining a core set of common genes throughout the brain [11,91]. The greatest overlap was found between cortical and NAc microglia, representing 84.3% of genes [11]. On the other hand, the most significant differences were observed in the VTA area, with VTA microglia presenting fewer genes involved in glycolysis and gluconeogenesis and genes involved in microglial function or oxidative phosphorylation [11]. The transcriptomic differences extend to the cerebellar or hippocampal level [100]. Compared to the cortex, the greatest variations were observed in the cerebellum in genes involved in various immune aspects, the hippocampus having an intermediate profile [101]. Thus, it was concluded that cerebellar microglia exist in a more immune vigilant state with increased expressions of antiviral interferon networks or genes involved in pathogen (or self-) recognition [101]. This increased vigilance may be influenced by local differences in the physical and neurochemical environment but also by blood–brain barrier permeability and neurotransmitter profiles [101]. The expression of genes involved in energy production was also increased; cerebellar microglia, due to their lower density, scan a larger volume of cerebellar parenchyma and, therefore, have higher energy needs [101].

## 5. Microglia States: From Surveillant to Reactive

**Figure 2:**
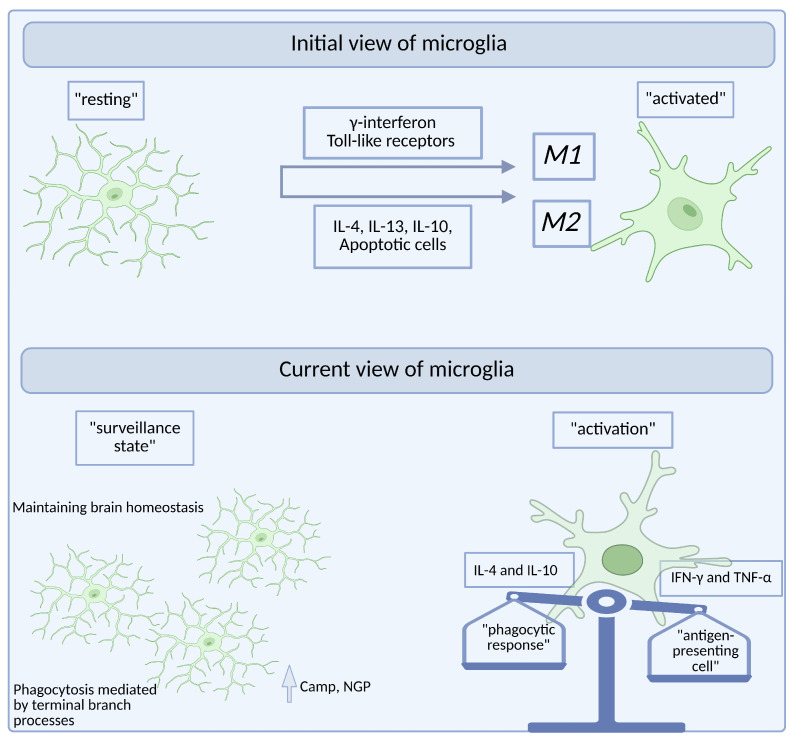
Conceptual shift in microglial phenotypes: from “resting vs. activated” to a functional continuum. The figure illustrates the paradigm shift in defining the microglial phenotype. Previous studies were based on the old paradigm, which assumed a binary definition of microglia ranging from “resting” to “activated”. It has now been demonstrated that microglia previously considered resting have important roles in the CNS and that activation constitutes a spectrum of microglial responses, with the phagocytic response at one end and the antigen-presenting cell function at the other. Furthermore, it is not uncommon for microglia to produce a mixed response.

In the mid-1970s, microglia were classified into two states: a static, well-ramified form called “resting microglia” and “activated microglia” spotted in pathological conditions and exhibiting a rounded, amoeboid shape [9,19,21,102]. However, the introduction of in vivo two-photon microscopy has uniquely illustrated that “resting microglia” are highly dynamic cells that are constantly engaged in monitoring their environment [1,103,104]. The paradigm shifts are represented in Figure 2, with surveillant microglia having an essential role in maintaining brain homeostasis. As such, the term “resting microglia” has slowly been replaced by “surveillant microglia”, although it has been reported that in this “surveillant state”, microglia are capable of phagocytosis through their terminal branches [103]. This is because rather than just responding to endogenous/exogenous activating factors, microglia actively maintain CNS homeostasis by sensing small shifts in the microenvironment, such as changes in neurotransmitters or neurotrophic factors [105]. As such, the “surveillant” microglia, without completely changing their state, can exhibit high levels of antimicrobial peptides, such as Camp (cathelin-related antimicrobial peptide) and Ngp (neutrophilic granule protein), and therefore play an important role in immune defense [106].

Furthermore, microglia activation, although tightly regulated, can occur as a response to a plethora of signals. Bacterial components like LPS or viral genetic material are considered external triggers, the so-called pathogen-associated molecular patterns (PAMPs) [107,108]. Internal signals, including nucleotides or misfolded proteins like amyloid β (Aβ) plaques, are also released by stressed or injured cells and can activate microglia [7]. These factors signal a drastic transition in the microglial state, which is considered to facilitate phagocytosis and promote repair [109]. A notable example is brain ischemia, where after 24 h of a transient focal cerebral ischemia, microglia exhibit smaller cell perimeters, with a shorter length in their overall branch tree and greater circularity of the soma [25]. This is followed by an increase in microglial movement toward the lesion [24]. At the molecular level, activated microglia, previously classified into M1 and M2 types, are characterized by the production of inflammatory cytokines and chemokines such as tumor necrosis factor-alpha (TNF-α) [110], interleukin (IL)-6 [111], as well as anti-inflammatory cytokines (IL-10, transforming growth factor (TGF)-β) [112,113], and growth factors like insulin-like growth factor-1 (IGF-1) [114]. Concomitantly, the surface proteins expressed by microglia change. Higher levels of CD16, CD32, CD40, CD86, or MHC II have been reported in the activated state [56]. Their importance resides in the fact that they are the first line in pathogen recognition, and their activation triggers a cascade of intracellular changes, controlling proliferation, differentiation, phagocytosis, and cell motility [56].

Just as the definition of microglia in the resting state has undergone changes over time, the same is evident in the context of activation [115]. If activated microglia were previously highlighted as a single phenotype—namely, they were characterized by the adoption of an amoeboid morphology [116]—subsequent research has highlighted the existence of a continuum of microglial activation states [117]. These go beyond the binary M1/M2 distinctions [118] and propose more of a spectrum with the phagocytic response at one end and the antigen-presenting cell function at the other [117]. Those that determine these varied responses are represented by the variety of external stimuli, for example, the stimulation of toll-like receptors produces a mixed microglial response, leading, on the one hand, to increased secretion of proinflammatory molecules and, on the other hand, to possible activation of T lymphocytes [117]. Furthermore, in neurodegenerative diseases, where there seems to be a gradual shift in between the two states, the changes are not only cellular [119] but also molecular, with the distribution and types of membrane channels being gradually shifted [120], and, as such, exerting a huge impact on their function [120,121].

## 6. Age-Related Microglial Changes

**Figure 3:**
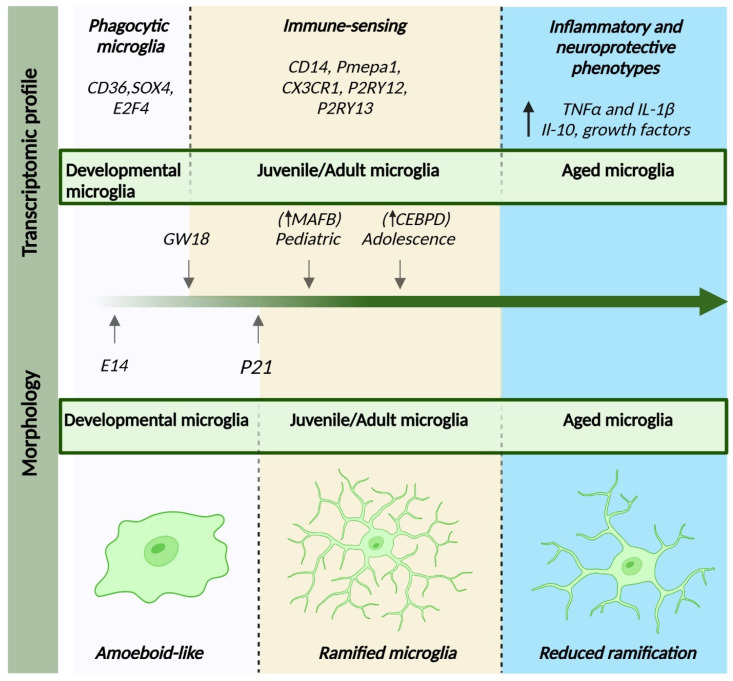
Morphological and transcriptomic profiles of microglia across the lifespan. The data shown represent normal aging in both the mouse and human brain, combining morphological and transcriptomic findings from both species. The black arrows indicate an increase in the corresponding gene expression. We have classified microglia into three stages: developmental microglia, which exhibit an amoeboid-like morphology and show upregulation of genes related to phagocytosis and cell cycle [16,122]. Juvenile/adult microglia begin to display their adult-like morphology around P21, with transcriptomic changes emerging as early as GW18, reflecting their maturation toward synaptic pruning and immune surveillance [16]. Aged microglia differ from both developmental amoeboid-like microglia and activated microglia; they exhibit a less ramified morphology and show upregulation of genes involved in pathogen recognition and defense, alongside increased expression of neuroprotective genes [12,106].

Microglia development seems to be characterized by three distinct stages: early microglia (before E14), followed by pre-microglia and adult microglia [122]. In the early stages, microglia express genes involved in the cell cycle and in cell differentiation while also exhibiting substantial phagocytic activity, which is crucial for regulating the activity of the neural progenitor cells [16,122,123]. Unsurprisingly, during this stage, microglia present with an amoeboid-like form [11]. Moreover, early postnatal white-matter-associated microglia [124] exhibit high heterogeneity when compared to the transcriptional profiles of adult microglia [125]. Some of their characteristics are also thought to be similar to the disease-associated microglia but with distinct biological functions. They possess elevated expression of genes linked to phagocytosis and lipid metabolism (e.g., *Gpnmb*, *Spp1*, *Clec7a*) and have an important role in myelination by engulfing newly formed oligodendrocytes [125]. This might be why, during this “juvenile” period, the morphology is hyper-ramified compared to adult microglia (Figure 3).

Transcriptomic studies show a slight deviation between protein expression and morphology. Prenatal microglia acquire a mature phenotype by gestational week 12 [126] and continue to develop until GW18, at which point, they achieve an immune-sensing phenotype [14]. Independent of the protein changes, morphological characteristics that bring them closer to their adult appearance seem to be generated in a distinct manner. The thinner processes begin to emerge at P7, and by 3 weeks, microglia resemble the adult form closely [12,88]. At this age, microglia already present a rich arborization, with branches that successively distribute up to the terminal ones and with a mature cellular domain [12,87]. The microglial population reaches high densities in the early postnatal period [12], which corresponds to the maturation phenomenon that characterizes the entire brain in the attempt to build optimal neuronal circuits [127]. Later, in a so-called “young adult” stage, a marked decrease in the microglial population is observed [12], with correspondence at the transcriptional level in the “pediatric” microglia, where the most expressed transcription factor remains *MAFB* (a protein coding gene which is considered to limit proliferation) [16]. The same study identified *CEBPD* as having a marked expression during adolescence [16], which defines an increased level of microglia activity during this period, as the gene is involved in the regulation of lipid metabolism or, rather, promotes lipid accumulation at the macrophage level [128].

The morphological and transcriptomic changes observed in microglia in the normal aging process are depicted in Figure 3.

As previously iterated, the appearance of the cell is closely connected to its function. A subtype of microglia found in the adult brain is defined by a rod-shaped morphology (a narrow, elongated cell body with polarized processes) [33]. More than a century ago, these microglia were initially associated with infections like typhus and syphilis [33], but nowadays, in the era of antibiotics, they are especially seen in the hippocampus of aged individuals, without relation to cognitive impairment [31]. Although they have an atypical appearance for microglia, their precise function is still under investigation. Studies suggest they may act as a barrier to protect uninjured neurons from a toxic microenvironment, acting as a stopper for damaging elements [32].

Age-related morphological changes in microglia include a reduced number of processes and reduced branch length and arbor area [12,24,129,130]. The effectiveness with which microglia scan their microenvironment is related to the arbor area but also determined by the dynamics of the process movement, which is considered to decrease in aged animals [24]. However, when it comes to the soma movement, a process seen in activation and not as a function of surveillance, increased values were obtained in the aged group [24]. More activation-related responses are considered to be affected in aging mice, this time via the movement of processes toward the lesion site or the activated-induced thickness of the processes, which are thought to decrease [24]. The same applies to the microglial transcriptomic profile, with a downregulation of genes related to endogenous ligand sensing and an upregulation of those involved in pathogen recognition and defense, alongside increased expression of neuroprotective genes [106]. This can be considered an alternative phenotype [106] that is diverging from the microglial priming state and the proinflammatory status that was considered as characteristic of the aging brain [131].

Another subtype identified is that of “dystrophic microglia”, which show a fragmented (but ultrastructurally intact) spheroid formation and increased tortuosity, distinct from activation-associated changes [28]. These features are different from the ones seen in activation and are indicative of aging and cellular senescence [28]. Moreover, dystrophic microglia can be considered a subtype of disease-associated microglia, since these changes are commonly observed in neurodegenerative diseases, and their presence is associated with age-related cognitive decline, akin to the senescent changes in neurons [29].

## 7. Microglia in Disease

Global population aging has become a significant challenge, not merely through the problems inherent in longevity but also by increasing the disease burden [132]. Neurodegenerative diseases have aging as a common risk factor, and although each of them has its own etiology, the role of microglia in their pathogenesis is being increasingly studied [133,134].

Given the limited availability of human pathological data, especially in the early stages of disease, reliance on animal models has become increasingly important. Murine models have managed to define the disease-associated microglial phenotype, filling the existing gap in human studies.

Initial studies were characterized mainly by morphological determinations, highlighting changes in microglia that respond through an activated amoeboid form and by clustering in the vicinity of amyloid plaques [135,136]. Furthermore, microglia in plaque-free cortical areas show a lower degree of activation [137]. The presence of amyloid peptides with their aggregation in the form of amyloid plaques can itself lead to microglial dysfunction [138], with a reduction in phagocytic capacity, a term called “frustrated phagocytosis” [139]. Other characteristics of plaque-associated microglia include the increase in “spontaneous” calcium signals (a phenomenon also found in normal senescent microglia unrelated to plaque), with these microglia not being able to reliably respond to extracellular ATP signaling [140].

On the other hand, this microglial dysfunction may be potentiated by the aging phenomenon itself. Age-induced changes at the microglial level are present, and even if new transcriptomic studies show an increase in the expression of neuroprotective genes [106], most of the literature confirms a proinflammatory status that is highlighted in the aged microglia [141,142]. The term “inflammaging” describes precisely this phenomenon by which microglia chronically show increased expressions of proinflammatory markers, which can lead to alterations of the surrounding environment and can act as a “first hit” and favor the appearance of pathology [131]. Moreover, a reduction in the basic motility of senescent microglia, and thus a reduction in the scanning of the entire cerebral parenchyma or a reduction in the capacity of phagocytosis, even in the absence of amyloid plaques, characterizes the aging brain [143]. Age-induced microglial changes may result in the accumulation of β-amyloid, which, in turn, may potentiate microglial dysfunction, closing the vicious circle.

Transcriptomic studies have brought significant benefits to the study of neurodegenerative diseases. They have led to the discovery of a type of microglia characterized by increased phagocytosis, the disease-associated microglia (DAM) or microglia neurodegenerative phenotype (MGn), which have been identified in the context of AD, ALS, and aging [106,144]. This activity is dependent on Trem2, which means that the absence of this gene (or its variants) may affect phagocytosis in the late stages of the disease [144]. In addition to Trem2 loss of function, another example is Lpl, whose mutations are associated with an aggressive form of Alzheimer’s or the ApoE 4 allele [144]. Elevated levels of CD33 correlate with an increased risk of AD as a result of reduced microglial phagocytic activity [138].

Regarding the study of amyotrophic lateral sclerosis (ALS), a great role in investigating microglial activation was attributed to the SOD1 (G93A) mouse model, with evidence suggesting that microglia from the motor cortex possess a reduction in branching complexity and, implicitly, a phenotype similar to activation [145,146].

In Parkinson’s disease, neurons in the SNc, crucial for dopamine production, are particularly vulnerable to degeneration [147]. This region is characterized by a lower density of microglia, which may contribute to the increased susceptibility of SNc dopamine neurons to degenerative diseases, but also by microglia with an increased proinflammatory profile [11].

Until now, the agreed therapeutic strategy in neurodegenerative diseases, which are characterized by neuronal loss and degeneration, was the target of neurons and neuroplasticity [148]. With the present advances in the understanding of microglial functions, particularly by utilizing disease-specific profiles, targeting microglia is considered a key to modulating neuroinflammation. The most enriched transcript in microglia, associated with Sandhoff disease, is the enzyme Hexb, suggesting the involvement of microglia in this neurodegenerative disease and its potential as a therapeutic target [149]. Another possible therapeutic target is represented by the TREM2-APOE pathway, which is considered a major regulator of the microglial phenotype in neurodegenerative diseases [150].

## 8. Conclusions

Transcriptomic profiling provides an in-depth view of gene expression changes, identifying potential therapeutic targets by highlighting genes enriched in microglia or those whose upregulation or downregulation alters processes like phagocytosis, neurogenesis, or synaptic pruning. Even though the morphological approach may be considered old-fashioned, it provides important details in microglial characterization and should be used as a complementary analysis to the transcriptomic approach. The morphological observation of microglia provides notable information such as the ability of clustering near Aβ plaques or the development of the microglial scar in the case of injury. Even slight adjustments in their density can offer important clues regarding activation. This continues with single-cell analysis, where assessing parameters such as the nucleus area or the degree of arborization can help us identify different microglial populations with distinct roles in homeostatic conditions and disease. Moreover, this enables real-time monitoring of microglial changes, whereas transcriptomic analysis typically reflects more stable, long-term gene expression alterations and may miss transient or subtle shifts in microglial phenotypes that occur over shorter timeframes.

## 9. Future Directions

The boost brought about by the 21st century in microglia publications reflects the increasing recognition of microglia’s pivotal roles in brain function and pathology. The body of research also grows more diverse with hundreds of measured parameters that claim to capture as close as possible the different microglia states. The main problem remains reproducibility across studies and the attempt to interpret the existing findings in the context of functional heterogeneity. In the future, the development of standardized pipelines or a shared database of protocols could greatly enhance reliability and statistical power.

## Figures and Tables

**Table 1:** Morphological diversity of microglia. This table summarizes the morphological phenotypes of microglia. The general aspects of microglial morphology are provided below, along with key references that offer further insights into the characteristics of each phenotype.

Microglial Phenotypes	General Aspect	Publications
Ramified microglia 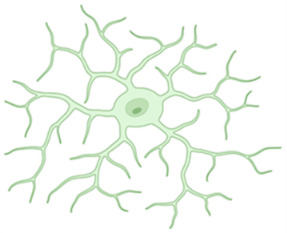	A small number of primary branches dichotomize up to the level of the terminal branches, reaching a complex arborization.	[12,19,24]
Amoeboid microglia 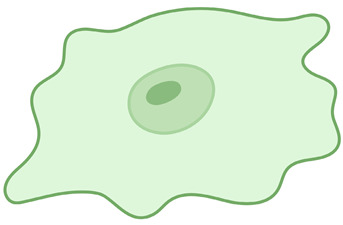	Can be considered precursors to activation; display the smallest values for general morphological parameters. They are also found in regions with an incomplete BBB, such as the median eminence, the circumventricular organs, and the subventricular zone.	[25,26]
Activated microglia 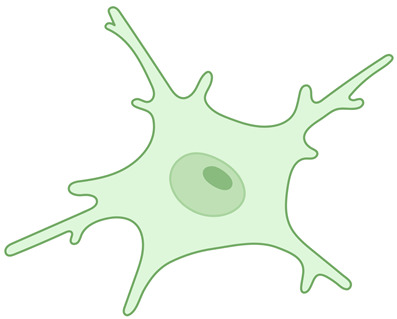	Have a smaller branching index, smaller cell perimeters, greater circularity of the soma, and cytoplasmic hypertrophy.	[19,24]
Aged microglia 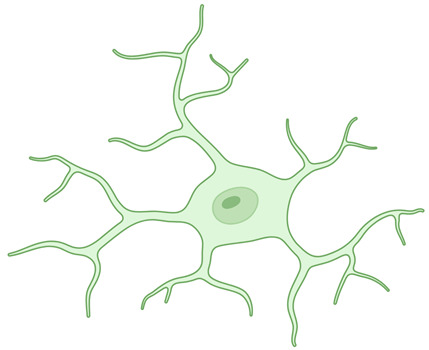	Reduced process length, branching, and arborized area. They possess reduced baseline process motility but increased soma motility. Upon activation, a reduced speed of microglial processes approaching the lesion was observed.	[12,24,27]
Dystrophic microglia 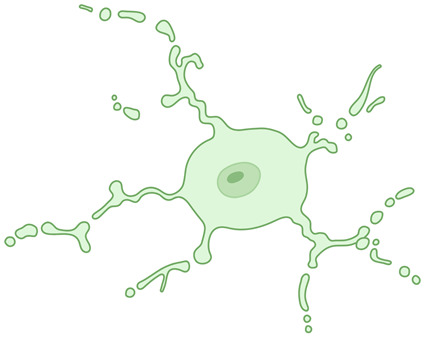	Fragmented and beaded processes, increased tortuosity, swellings distinct from activation. They have been encountered in the aged brain, and furthermore, in the case of neurodegenerative disorders, are considered the morphological expression of disease-associated microglia.	[28,29]
Rod microglia 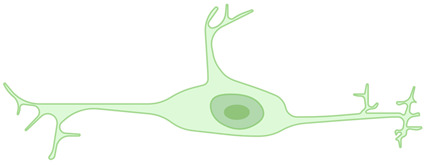	A narrow, elongated cell body with polarized processes. They are currently considered a particular form of microglial activation, occurring only in diseases affecting the CNS.	[30,31,32,33,34]

## Abbreviations

The following abbreviations are used in this manuscript:

scRNA-seq	Single-cell RNA sequencing
AD	Alzheimer’s disease
CXCL12	C-X-C motif chemokine 12
CCL2	Linear dichroism
GS-1	Griffonia simplicifolia 1
RCA	Ricinus communis agglutinin
WGA	Wheat germ agglutinin
ConA	Concanavalin A
F4/80	EGF module-containing mucin-like receptor
CD40	Cluster of differentiation 40
CD11b	Integrin αM subunit
CD80	Cluster of differentiation 80
TREM2	Triggering receptor expressed on myeloid cells 2
Iba1	Ionized calcium-binding adaptor molecule 1
CD68	Cluster of differentiation 68
ICAM-1	Intercellular adhesion molecule-1
CX3CR1	C-X3-C motif chemokine receptor 1
Tmem119	Transmembrane protein 119
Hexb	Hexosaminidase subunit beta
HFD	High-fat diet
NAc	Nucleus accumbens
SNr	Substantia nigra pars reticulata
VTA	Ventral tegmental area
SNc	Substantia nigra pars compacta
CA3	Hippocampal cornu ammonis
CA1	Hippocampal cornu ammonis
Camp	Cathelin-related antimicrobial peptide
Ngp	Neutrophilic granule protein
PAMPs	Pathogen-associated molecular patterns
Aβ	Amyloid β
TNF-α	Tumor necrosis factor-alpha
IL-6	Interleukin-6
IL-10	Interleukin-10
IL-13	Interleukin-13
IFN-γ	Interferon-γ
TGF-β	Transforming growth factor-β
IGF-1	Insulin-like growth factor-1
CD16	Cluster of differentiation 16
CD32	Cluster of differentiation 32
CD40	Cluster of differentiation 40
CD86	Cluster of differentiation 86
MHC II	Major histocompatibility complex Class II
E14	Embryonic day 14
*Gpnmb*	*Glycoprotein nonmetastatic melanoma protein B*
*Spp1*	*Osteopontin*
*Clec7a*	*C-type lectin domain family 7*
GW	Gestational week
P7	Postnatal day 7
*MAFB*	*Musculoaponeurotic fibrosarcoma oncogene homolog B*
*CEBPD*	*CCAAT/enhancer-binding protein delta*
ATP	Adenosine triphosphate
DAM	Disease-associated microglia
MGn	Microglia neurodegenerative phenotype
Trem2	Triggering receptor expressed on myeloid cells 2
Lpl	Lipoproteinlipase
CD33	Sialic acid-binding Ig-like lectin 3
ApoE	Apolipoprotein E
SOD1(G93A)	Superoxide dismutase-1 glycine 93 to alanine
ALS	Amyotrophic lateral sclerosis
BBB	Blood–brain barrier
CVOs	Circumventricular organs
*CD36*	*Platelet glycoprotein 4*
*SOX4*	*Sex-determining region Y-related high mobility group-BOX gene 4*
*E2F4*	*E2F Transcription Factor 4*
*CD14*	*Cluster of differentiation 14*
*Pmepa1*	*Prostate transmembrane protein androgen induced 1*
*P2RY12*	*Purinergic Receptor P2Y12*
*P2RY13*	*P2Y purinoceptor 13*
*IL-1β*	*Interleukin-1β*
